# Graph neural network-based cell switching for energy optimization in ultra-dense heterogeneous networks

**DOI:** 10.1038/s41598-022-25800-3

**Published:** 2022-12-14

**Authors:** Kang Tan, Duncan Bremner, Julien Le Kernec, Yusuf Sambo, Lei Zhang, Muhammad Ali Imran

**Affiliations:** James Watt School of Engineering, University of Glasgow, Glasgow, UK

**Keywords:** Electrical and electronic engineering, Computational science

## Abstract

The development of ultra-dense heterogeneous networks (HetNets) will cause a significant rise in energy consumption with large-scale base station (BS) deployments, requiring cellular networks to be more energy efficient to reduce operational expense and promote sustainability. Cell switching is an effective method to achieve the energy efficiency goals, but traditional heuristic cell switching algorithms are computationally demanding with limited generalization abilities for ultra-dense HetNet applications, motivating the usage of machine learning techniques for adaptive cell switching. Graph neural networks (GNNs) are powerful deep learning models with strong generalization abilities but receive little attention for cell switching. This paper proposes a GNN-based cell switching solution (GBCSS) that has a smaller computational complexity than existing heuristic algorithms. The presented performance evaluation uses the Milan telecommunication dataset based on real-world call detail records, comparing GBCSS with a traditional exhaustive search (ES) algorithm, a state-of-the-art learning-based algorithm, and the baseline without cell switching. Results indicate that GBCSS achieves a 10.41% energy efficiency gain when compared with the baseline and achieves 75.76% of the optimal performance obtained with ES algorithm. The results also demonstrate GBCSS’ significant scalability and generalization abilities to differing load conditions and the number of BSs, suggesting this approach is well-suited to ultra-dense HetNet deployment.

## Introduction

Since 2010, there has been a proliferation of mobile phones and Internet of Things (IoT) devices, and the development of advanced mobile applications with differing Quality of service (QoS) requirements for the user. This has resulted in a dramatic increase in demand for mobile services leading to a significant increase in base station (BS) deployment density of differing types and capabilities to meet the network demand, improve coverage, and support a multitude of mobile applications, leading to the formation of ultra-dense heterogeneous networks (HetNet). However, such proliferation has significantly increased the environmental and economical burden on society. Environmentally, the information and communications technology (ICT) sector must reduce its CO$_2$ emissions by 42% by 2030 and 72% by 2040 in line with other sectors to stay within the 1.5 °C global goal^1^. Additionally, the economical burden of energy cost absorbs between 15% and 50% of the total cellular network operational expenses in mature and developing markets respectively^2^. These changes all require improvements in network energy efficiency towards green and environmentally sustainable radio access networks, which will also deliver benefits through the reduction of operational expense (OpEx) reduction while ensuring the mobile services.

BSs are the major energy consumers in cellular networks and account for 60% to 80% of cellular network’s total power consumption^3^. With the development of green radios, different kinds of energy efficiency schemes have been proposed, such as engineering higher-efficiency power amplifiers and reducing the transmit power while keeping service QoS via efficient scheduling, etc.^4^ However, the conventional strategy has been to maintain constant BS operation even when no active users are using the BS’s coverage, resulting in significant energy wastage. As traffic loads of cellular networks show both temporal and spatial variation, load adaptive network operation can also be executed such that BSs could be switched off or to operate in low power modes during periods of low /no traffic to optimize the energy efficiency, forming another type of power efficiency scheme. However, it may not always be feasible to completely switch off BSs in the cellular network architecture due to potential coverage holes that would inevitably downgrade the users’ experience(QoS). Moreover, a sleeping BS cannot transmit signals needed by users equipment (UEs) to establish connections such as cell discovery and channel estimation^5^.

Separating the signals requiring full coverage from those supporting high data rate transmissions, the Control Data Separated Architecture (CDSA) is a crucial network architecture to the above challenge^5^. In CDSA, a macro cell control BS (MC) provides constant coverage, general data services, and handles signalling tasks, while small cell data BSs (SCs) provide high data rate services to support various mobile applications. By appointing an MC to ensure the service coverage and the backhaul connection between the MC and SCs, CSDA brings the possibility to switch SCs within the MC’s cover into deep sleep mode without impacting the users’ QoS during cell switching operation and traffic load re-association.

Research has been conducted for optimized cell switching solutions in CDSA HetNets, and analytical models and heuristic algorithms were developed with *a priori* knowledge of the environment^6–8^. However, such approaches usually face the NP-hardness solving issue due to the problem formation complexity and computational overhead for complex scenarios, and have limited generalization capability adapting to the dynamic environment of wireless networks^9,10^.

In comparison, machine learning (ML) techniques are able to extract knowledge from historical and real-time collected data for cell switching decision optimization. Reinforcement learning (RL) based algorithms can directly optimize cell switching strategies^11–14^ while other ML techniques for prediction, classification, and clustering are capable of assisting cell switching solutions for improved performances^15–17^. Furthermore, deep learning techniques utilizing the strong approximation capability of artificial neural networks (ANNs or NNs) can accommodate highly complex scenarios by directly learning patterns from the rich datasets generated by the communication networks.

In recent years, graph neural networks (GNNs) has received much attention from the research community, with GNN’s strong expressive power and generalization ability achieved successes in different research areas such as in computer vision, chemistry, and social networks^18,19^. It has also been applied to wireless network research including traffic prediction, power control, etc.^20^, as communication networks can be naturally modelled using graphs. Compared to existing deep learning-based solutions, GNNs show an advantage of better generalization capability through learning the network topology via the graph data structure with node size invariance^21,22^. The ability of learning the underlying topology on graph structured data can increase dataset utilization efficiency and the learning robustness, while node-size invariance is a significant advantage that reduces computational and time cost for retraining time after deployment to differing scenarios when compared to other deep learning techniques such as deep RL, which needs retraining when the action space size changes. Both advantages make GNN a powerful candidate for cell switching decision optimization. However, little research has been conducted exploring how GNNs perform in cell switching problems to date.

This paper focuses on a first attempt to develop a GNN-based cell-switching solution for CDSA HetNets that can be deployed at each MC of the network to provide cell-switching decisions for SCs within its coverage at a system-level in a locally centralized manner. The proposed solution consists of the graph representation of individual HetNet units, GNN computational model building, and loss function design for unsupervised training. The performance of the proposed solution is evaluated using a dataset based on real-world cellular network traffic information. The performance results are compared to the theoretical optimal results calculated by the exhaustive search algorithm, a state-of-the-art RL-based solution, and the All-on method representing no cell switching deployment. Note that although high-level discussions on how the proposed cell switching algorithm may be deployed in the cellular protocol stack, this work focuses on the algorithmic development and the detailed deployment aspect is beyond the scope of this work. The contributions of this work are summarized as follows:

- A graph representation of a CDSA HetNet unit considering BSs’ traffic loads and power consumption, and a GNN-based cell-switching solution (GBCSS) for CDSA HetNets. GBCSS has a much lower computational complexity during execution compared to the ES algorithm hence is scalable and tractable for large deployments for 6G super connectivity.
- The proposed GBCSS is evaluated using a well-established telecommunication dataset^23^ that is based on real-world call detail record (CDR) information in the city of Milan, making the results more realistic.
- Evaluation results show a 10.41% power efficiency gain using the GBCSS with respect to the baseline without cell switching. Compared to the ES algorithm used for upper bound baseline, the GBCSS achieves 75.76% of the optimal performance results with less than 0.5% of user QoS sacrificed. In addition, the average energy efficiency of GBCSS outperforms that of the other learning-based benchmark algorithm by 11.90%.
- Generalization tests for different date, time, and node size show the GBCSS’ strong generalization ability that makes the method highly promising for practical deployments.

## Related work

Cell-switching decision optimization must find the best combination of SCs to offload traffic and switch off in order to maximize power saving while maintaining user QoS. Such a problem is naturally combinatorial and may be formulated as mixed integer programming with multiple sets of variables to consider trade-offs among metrics^3^ as too aggressive cell switching may lead to user QoS sacrifices when maximizing power saving, while too mild cell switching leads to service capacity beyond users’ demands and still causes energy wastage. Various approaches exist in the literature to implement cell switching optimization in CDSA HetNets for energy optimization. These methods can be broadly classified to heuristic algorithms and ML-based direct cell switching decision-making (mainly RL-based). Some research also developed multi-tier solutions combining heuristic algorithms with ML methods, or developing combinations of different ML methods (e.g. supervised learning and RL).

For heuristic algorithms, the exhaustive search (ES) algorithm ensures to produce the optimal cell switching results by traversing the whole search space to find the best SC combination(s) based on the objectives while satisfying the constraints. However, the complexity of this algorithm grows exponentially and is only practical to apply to small search spaces^12^. To improve the search efficiency towards an optimal solution, a suboptimal greedy SC on/off strategy was proposed in^6^ to determine the SC switching patterns for a BS cluster in a green ultra-dense HetNet. This greedy heuristic algorithm tried to maximize the network energy efficiency while considering traffic load of the SCs and user QoS requirements. Similarly, a firefly algorithm was developed in^7^, where joint optimization of the area spectral efficiency and energy efficiency was formulated to determine the optimal system parameters for a two-tier ultra-dense HetNet. Moreover, a cooperative energy optimization scheme for 5G ultra-dense HetNet using graph theory was proposed in^8^, where a graph representation of the network was first developed, followed by applying graph theory to determine the order of SC nodes to which power-off/on procedures are applied.

Heuristic algorithms are hard-coded with limited generalization ability, and recurrent applications are often required when network conditions change significantly. To tackle such challenges, some recent research developed ML-based solution. For example, a dynamic SC load adjustment algorithm was proposed in^11^ that used Q-learning to train an optimal offloading and load-balancing policy to switch off redundant SCs in an ultra-dense HetNet. A distributed Q-learning technique was utilized in^13^ that modelled each SC as an agent jointly learn to choose the best sleep modes in a multi-sleep-mode HetNet setup, in order to maximize the network’s energy efficiency. However, such tabular RL methods require a large state-action table (or Q-table) to represent the optimal policy when the HetNet scale rises, which leads to considerable memory consumption. As a results, approximation-based RL algorithms become a promising candidate, such as the SARSA algorithm with linear function approximation proposed by Ozturk et al.^12^. Deployed for online training and execution in an ultra-dense HetNet, the feature space of the SARSA algorithm contains all BSs’ traffic loads with the total network-wise power consumption for optimal binary cell switching policies for SCs. Another approach is to exploit the strong approximation capability of ANNs, such as the work of Zhang et al. who developed a double deep Q-network to determine the optimal sleeping strategy in a heterogeneous radio access network^14^. The algorithm was trained and tested using real-world traffic data to minimize the energy consumption of the HetNet while maintaining the user QoS within the network.

If multi-tier solutions are considered, some research combined ML methods to boost the performance of heuristic algorithms, or to reduce the problem search space and hence the overall problem complexity. Abubakar et al. proposed a two-tier cell switching based on unsupervised learning and the ES algorithm^15^. Their solution first separated an ultra-dense HetNet into different clusters using the K-means algorithm, after that the ES algorithm was executed for each cluster to get optimal local cell-switching decisions. A long short-term memory recurrent NN (RNN) model was utilized by Jang et al.^16^ to predict user traffic for the next few time slots of the network. Based on the predicted traffic, a Lyapunov optimization problem was formulated to obtain the cell-switching decision to balance between the reduced power consumption and the predicted traffic loads.

Moreover, different learning-based techniques can be jointly utilized for cell switching decision making, such as the work in^17^ that first combined convolutional NN and RNN to leverage the geographical and semantic spatial-temporal correlations of mobile traffic for future traffic prediction. After that, the cell switching problem was modelled as a Markov decision process and solved by the deep deterministic policy gradient method, a deep RL algorithm.

Different learning-based solutions have been proposed in the literature for cell switching optimization, while GNN techniques received little attention although with strong expressive capability and explored to be effective in solving a similar problem of link scheduling^22,24^. For instance, Lee et al.^24^ proposed a graph representation design for device-to-device communication and utilized graph embedding combined with neural networks to learn an optimal link scheduling decision without requiring channel state information. Their performance results showed that graph representation learning is competitive in performance optimality, generalization ability and scalability. However, their design centered around communication links cannot be directly adapted to the cell switching use case, which also motivated our work to explore GNNs on cell switching.

Following the advantages covered in Introduction, GNN has the following advantages over other learning-based techniques for the cell switching problem: GNN learns on graph-structured data, which include relationship information among modelled BSs, which is absent from other techniques while being useful to the NN model to learn the features with extra information and hence reduce the training epochs; GNN is capable of being extended to different-sized network without the need of retraining, which considerably reduces the cost for deployment to different HetNets compared to other learning-based techniques such as deep Q-learning.

Consequently, this work in our paper chose to explore a GNN-based optimal cell switching solution using unsupervised training approach. The proposed solutions consists of a graph representation of the considered system model, followed by GBCSS, an end-to-end GNN offline training and online execution design. The performance of the proposed GBCSS is evaluated using real-world traffic data to generate more realistic results.

## System model and problem formulation

### Network architecture and power consumption model

**Figure 1:**
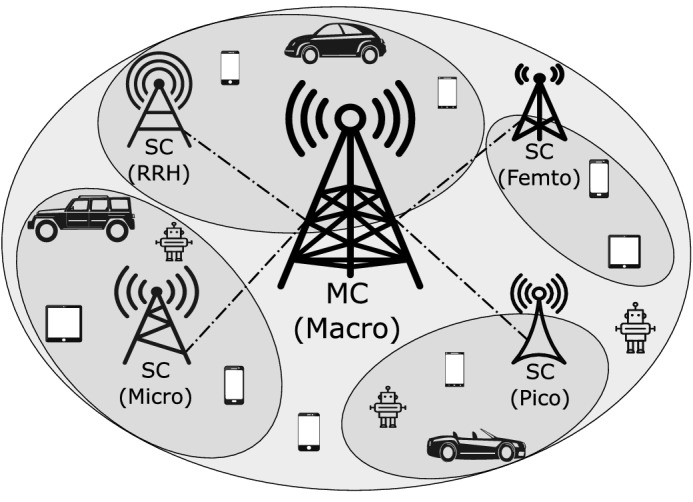
A CDSA HetNet unit consisting of a MC and densely deployed SCs within the coverage of the MC, SCs can be of type Remote Radio Head (RRH), Micro, Pico, and Femto BSs according to^25^.

This work considers an ultra-dense HetNet with a CDSA architecture^5^, formulated by multiple HetNet units comprising of one MC and multiple SCs of different types within the coverage of the MC. For each HetNet unit as shown in Fig. 1, the MC serves as the control BS for signalling, and provides constant coverage and data services, while the SCs only handle data services based on user specific requests for network capacity enhancement. The MC also acts as a centralized controller within its coverage area for switching SCs in/out of sleep mode and for traffic offloading. This task contains traffic load observations on all local SCs, and decision making on the set of SCs that should be switched into sleep mode during periods of low traffic intensity, with the available capacity of the MC also taken into consideration.

Using the above system model, the instantaneous power consumption of a HetNet unit $P_{tot}$ containing 1 MC and $N_{SC}$ SCs (SCs’ BS types implied) in a CDSA HetNet is given by:

1 $$\begin{aligned} P_{tot} = \sum _{i = 1}^{N_{SC}+1}P_{BS}^{i} =P_{MC} + \sum _{i = 1}^{N_{SC}}P_{SC}^{i} \end{aligned}$$

where $P_{BS}^{i}$ is the power consumption of the *i*th BS ($BS_i$) in the HetNet unit, BS type implied, while $P_{MC}$ and $P_{SC}^i$ denote the power consumption of the MC and the $i^{\textrm{th}}$ SC respectively.

Derived from the Energy Aware Radio and neTwork tecHnologies (EARTH) power consumption model^25,26^, the calculation of $P_{BS}^{i}$ for every BS type is expressed as:

2 $$\begin{aligned} P_{BS}^{i} = \left\{ \begin{aligned} P_o^i + \Delta _p^i P_{tx}^i, \ \ 0&< P_{tx}^i \le P_{max}^i \\ P_s^i, \ \ \ \ \ \ \ \ \ \ \ \ \ \ \ P_{tx}^i&= 0 \\ \end{aligned}\right. \end{aligned}$$

where $P_o^i$, $P_{s}^i$ are the static operational and sleeping power consumption respectively, and $\Delta _p^i$ is the slope of the load-dependent power consumption. $P_{tx}^i$ is the transmission power that is proportional to the maximum transmission power $P_{max}^i$ based on a BS’s factorized traffic load, expressed as:

3 $$\begin{aligned} P_{tx}^i = \lambda _t^i P_{max}^i \end{aligned}$$

where $\lambda _t^i \in [0, 1]$ is $BS_i$’s factorized traffic load at time step *t*, defined as:

4 $$\begin{aligned} \lambda _t^i = \frac{d_t^i}{C_i} \end{aligned}$$

where $d_t^i$ represents the radio resources of $BS_i$ utilized by its served users at *t*, and $C_i$ is the radio resource capacity of $BS_i$.

It is also assumed that BSs of a given type (e.g. all micro BSs) are configured with identical hardware for this general problem formation, such that every type of BSs will have constant $P_o^i$, $P_{max}^i$, and $\Delta _p^i$. Therefore, $BS_i$’s power consumption $P_{BS}^{i}$ depends only on its traffic load $\lambda ^i$ and BS type. For real-world applications, values of these parameters can be specified based on individual BS setups.

### Problem formation

Following the above system model, the goal is to determine the optimal BS switching strategy (i.e. the optimal set of SCs to switch on/off) for each time slot *t* (in minutes) in a given time period ${\mathscr{T}}$ (in minutes), to minimize energy consumption while maintaining user QoS in a HetNet unit. The switching strategy at *t* is defined as $\Gamma _t = \{ \gamma _t^1, \gamma _t^2, \ldots \gamma _t^{N_{SC}+1} \}$, where $\gamma _t^i \in \{0, 1\}$ indicates the switching decision for $BS_i$ at *t*, 1 for ON and 0 for OFF. In this work, the MC of each HetNet unit is defined to be at index 1 ($BS_1$), and should be always be ON according to its functionality, i.e. $\gamma _t^1 = 1, \forall {t} \in T$.

By deciding the switching strategy $\Gamma _t$ at each time slot *t*, a traffic re-association stage is carried out before the cell switching execution, during which the MC takes the traffic loads from, or allocates traffic loads to an SC within its coverage if that SC was switched to sleep mode or brought back in service, i.e. moving $d_i$ from $BS_i$ to $BS_1$, where $i \ne 1$. However, as SCs and the MC may have different capacities, it is essential to consider such difference for traffic re-association when using the factorized traffic loads $\lambda$ during this process. To represent such capacity differences, $\phi _i$ is introduced as the ratio of $BS_i$’ capacity to that of $BS_1$ (the MC). Note that $\phi _1$ is always 1 as it means the MC’s capacity comparing to itself:

5 $$\begin{aligned} \phi _i = \frac{C_i}{C_1} \end{aligned}$$

Therefore, for $BS_i \, (i \ne 1)$, $\phi _i \lambda _t^i = \frac{C_i}{C_1} \times \frac{d_t^i}{C_i} = \frac{d_i}{C_1}$ represents the factorized traffic load of this BS with respect to the capacity of $BS_1$ (the MC), and the original factorized sum traffic load $\Lambda _t$ at time slot *t* before executing cell switching $\Gamma _t$ can then be defined as (6). Note that $\Lambda _t$ is based on the capacity of $BS_1$ (the MC):

6 $$\begin{aligned} \Lambda _t = \lambda _t^1 + \sum _{i=2}^{N_{SC}+1} \phi _i\lambda _t^i = \sum _{i=2}^{N_{SC}+1} \frac{d_i}{C_1} \end{aligned}$$

Moreover, each BS’ traffic load after re-association and cell switching can be calculated as follows, starting with $BS_1$ (the MC):

7 $$\begin{aligned} \hat{\lambda }_t^1 = \lambda _{t}^1 + \sum _{i = 2}^{N_{SC} + 1} {[}\gamma _t^i\phi _i\lambda _{t}^i - (1 - \gamma _t^i)\phi _i \lambda _t^i], \ \ \text {if} \ \gamma _t^i \ne \gamma _{t-1}^i \end{aligned}$$

and for all SCs (for $i \ge 2$):

8 $$\begin{aligned} \hat{\lambda }_t^i =\left\{ \begin{aligned}&0 + (1 - \gamma _t^i) \lambda _{t}^i, \ \ \text {if} \ \gamma _t^i \ne \gamma _{t-1}^i \\&\lambda _{t}^i, \ \ \text {else}\\ \end{aligned}\right. \end{aligned}$$

where $\lambda _t^i$ and $\hat{\lambda _t^i}$ are the traffic loads of $BS_i$ at time slot *t* before and after the execution of traffic re-association and cell switching.

Note that after the cell switching execution, $BS_i$’s power consumption $P_{BS}^i$ will also change to $\hat{P}_{BS}^i$ upon $\lambda _t^i$ changes to $\hat{\lambda }_t^i$. Following Eq. (2), $\hat{P}_{BS}^i$ is hence calculated as:

9 $$\begin{aligned} \hat{P}_{BS}^i = \gamma _t^i (P_o^i + \Delta _p^i \hat{\lambda }_t^i P_{max}^i) + (1 - \gamma _t^i) P_s^i \end{aligned}$$

The factorized sum traffic load after cell switching $\hat{\Lambda }_t$ of the HetNet unit is then defined as (10). It is noteworthy that $\hat{\Lambda }_t \le \Lambda _t$ as switching off SCs after the MC reaches its capacity ($\hat{\lambda }_t^1 = 1$) will lead to sacrifices of the original traffic loads:

10 $$\begin{aligned} \hat{\Lambda }_t(\Gamma _t) = \hat{\lambda }_t^1 + \sum _{i=2}^{N_{SC}+1} \phi _i \hat{\lambda }_t^i \end{aligned}$$

Denote $\hat{P}_{tot}$ as the energy consumption of the HetNet unit after executing $\Gamma _t$, the optimization objective is hence to choose an optimal $\Gamma _t$ for the HetNet unit to maximize the energy efficiency for all $t \in T$, i.e. to minimize $\hat{P}_{tot}$ while maximizing $\hat{\Lambda }_t$ (to maintain $\Lambda _t$ as much as possible and thus preserve the original user QoS) in the HetNet unit. Combining (1), (9), and (10) this optimization can be formulated as follows, with $N_{SC}$ independent variables ( $\gamma _t \in \Gamma _t$) and two constraints:

11 $$\begin{aligned} \min _{\Gamma _t} \ \hat{P}_{tot}(\Gamma _t)&= \sum _{i = 1}^{N_{SC} + 1} \hat{P}_{BS}^i = \sum _{i = 1}^{N_{SC} + 1} [\gamma _t^i (P_o^i + \Delta _p^i \hat{\lambda }_t^i P_{max}^i) + (1 - \gamma _t^i) P_s^i], \end{aligned}$$

12 $$\begin{aligned} \text {s.t.} \ \max _{\Gamma _t} \ \hat{\Lambda }_t(\Gamma _t)&= \hat{\lambda }_t^1 + \sum _{i=2}^{N_{SC}+1} \phi _i\hat{\lambda }_t^i, \nonumber \\ \hat{\Lambda }_t&\le \Lambda _t, \ \ 0 \le \hat{\lambda }_t^i \le 1. \end{aligned}$$

where Eq. (11) defines the optimization objective to minimize a HetNet unit’s power consumption $\hat{P}_{tot}(\Gamma _t)$ given a switching decision $\Gamma _t$ at time slot *t*. Equation Eq. (12) defines the optimization constraints where $\hat{\Lambda }(\Gamma _t)$ is defined by (10), which is calculated as the sum of all factorized loads of local BSs with respect to the MC’s capacity. $\hat{\lambda }_t^i$ denotes $BS_i$’s load factor after switching, as defined above.

Note that the optimization constraint $\max _{\Gamma _t} \ \hat{\Lambda }(\Gamma _t)$ has an upper bound of $\Lambda _t$ which is the original traffic load of the HetNet unit at every time slot before executing cell switching as discussed above. Moreover, the value of $\hat{\lambda }_t^i$ should be between 0 and 1 to not exceed a BS’s capacity at each time slot after switching following the definition of $\lambda$.

Although the formulated cell switching optimization problem appears to be not complicated, it is a min-max trade off problem that needs to consider both the MC’s and all SCs’ traffic loads within a HetNet unit, while also needing to take the power consumption of different SC types into account (e.g. 4 SC types are considered in the experiments covered in this work as in Table. 1), which is not directly presented in Eq. (11). Therefore, the search space for an optimal cell switching decision is much larger with a highly complex underlying scenario, especially for a large number of SCs. As $\Gamma _t$ is a discrete set of binary values, the defined min-max optimization is naturally combinatorial, with $2^{N_{Sc}}$ possible combinations for every time slot *t* for a given HetNet unit.

Such combinatorial optimization can be considered as a variation of the Knapsack problem, which is a well-known NP-hard^12,26,27^. The Knapsack problem considers a set of $N_{obj}$ indivisible objects with integer labels $id = 1, 2, \ldots, N_{obj}$. Each object is associated a real number value $v_i$ and a positive real number weight $w_i$. The goal of the problem is to select a subset of these objects to achieve a maximum sum value while the maintaining the total weight within *W* units, and the mathematical formulation of the problem expressed as: find $o_i$, such that

13 $$\begin{aligned}&\max \ \sum _{id=1}^{N_{obj}} o_{id} \cdot v_{id}, \end{aligned}$$

14 $$\begin{aligned}&\text {s.t.} \ \sum _{id=1}^{N_{obj}} o_{id} \cdot w_{id} \le W, \nonumber \\&o_{id} \in \{0, 1\}, \ \ id = 1, 2, \ldots, N_{obj}. \end{aligned}$$

where the objective function (13) can be directly related to the cell switching objective (11) after transforming the minimization in (11) to a maximization form by treating BSs’ power consumptions as negative values, with the binary Knapsack decision $o_{id}$ representing $\gamma _i$. As for the constraint, the $\hat{\Lambda }_t \le \Lambda _t$ part of (12) represents the Knapsack constraint in (14), while an additional maximization is added in the formulated cell switching problem, making the cell switching problem overall a variation of the Knapsack problem.

## Cell switching via graph representation learning

GNN models learn on data represented by the graph data structure. Formally, a graph $g = ({\mathscr{V}}_g, {\mathscr{E}}_g, {\mathscr{X}}_g, {\mathscr{A}}_g)$ is composed of a set of vertexes/nodes ${\mathscr{V}}_g$ and a set of edges/arcs ${\mathscr{E}}_g$ connecting pairs of nodes^28^. When the node pairs in *g* are unordered, *g* is referred to as an undirected graph, while ordered node pairs in *g* make it a directed graph. To enrich the graph *g*, additional node and edge information can be included in ${\mathscr{X}}_g$ and ${\mathscr{A}}_g$ respectively. Each node $v \in {\mathscr{V}}_g$ is associated with a node feature $x_v \in {\mathscr{X}}_g$, while an edge $(\overline{u, v})$ connecting a pair of nodes *u*, *v* $(u \ne v)$ holds an edge attribute $a_{uv} \in {\mathscr{A}}_g$. A graph is a powerful data structure to model a set of objects (as nodes) and their relationships (as edges).

Following the general GNN design pipeline^29^, this section presents the GBCSS from the following aspects: graph representation design, GNN computational model building, and learning task confirmation with loss function design.

**Figure 2:**
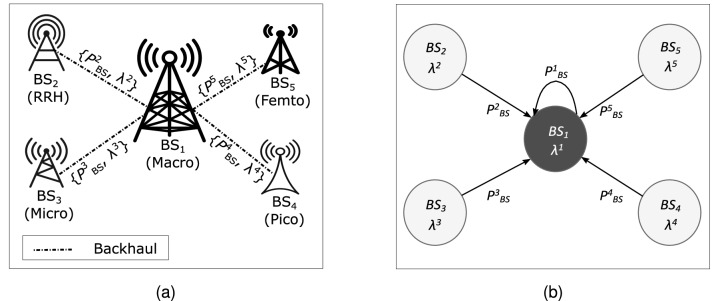
The proposed graph representation process (**a**) System model. (**b**) Graph representation model. Note that superscripts are for indexing purpose to match the notations in problem formation.

### Graph representation of a HetNet unit

Omitting the wireless communication links, the network architecture described in Fig. 1 can be expressed as the system model shown in Fig. 2a, where SCs within the MC’s coverage are connected to the MC through the backhaul. For cell-switching, each BS (SCs and the MC) contains the essential information regarding their current traffic load $\lambda$ and instantaneous power consumption $P_{BS}$, which is sent to the HetNet unit’s local controller located at the MC. Additionally, the type of each SC (e.g. micro or pico) should also be known by the local controller. For each time slot, the local controller decides the set of SCs to offload and switch off according to the received information, and then the MC sends the corresponding control signals to the SCs.

The proposed graphical modeling of the system model in Fig. 2a starts with treating each BS in the HetNet unit as a node, with the BS’s traffic load $\lambda$ modelled as the node feature $x_v$, while the power consumption $P_{BS}$ of each BS is treated as the edge attributes $a_{1,v}$ that connects the BS at node *v* and the MC at node 1. Following the above modeling, Fig. 2b demonstrates the proposed graph representation model. The neighbor design of this model is based on the system-level assumption that an SC sends it current load and power consumption data via the backhaul to the local controller deployed at the MC for cell-switching decision making. Note that the traffic loads and power consumption of the MC should also be sent to the local controller, hence another edge is added to the proposed graph design connecting the MC node to itself. Additionally, edges in this graph representation model are designed to be directed from each SC node to the MC node following the above information flow. For example, the edge connecting node $BS_3$ and node $BS_1$ has the direction of $BS_3 \rightarrow BS_1$, and this directed edge hence is denoted as $(\overrightarrow{BS_3, BS_1})$. Directionality reflects that different BSs have differing power consumptions based on the BS types and traffic loads. If an undirected graph representation is used, an edge feature is then shared by its connected node pair, which is not suitable to represent this differentiated power consumption and thus the relationship between an SC and the MC. Therefore, the directionality also allows distinct edge weights to be utilized by the graph convolutional operator introduced in the following section. The node and edge sizes of the proposed graph representation model are both identical to the total number of BSs within a HetNet unit (e.g. tens to hundreds) and thus denoted as *n*.

This graph representation should be considered as a dynamic graph; after cell switching, the state of all node and edge features change to $\hat{x}_v$ and $\hat{a}_{u,v}$, following the change of $\lambda$ to $\hat{\lambda }$ and the resultant $P_{BS}$ for all BSs calculated by Eqs. (7), (8), and (2). It should be recognized that other graph representation designs may have differentiated learning outcomes combined with different GNN models. However, investigating the performance of different modeling designs is beyond the scope of this paper.

### GNN computational model for cell-switching

The graph *g* serves as the underlying topology for a GNN backbone, and is taken as the GNN’s input. The GNN then learns and produces a state embedding for each node in *g*, containing the node’s own information and its neighborhood. Specifically, the GNN processes the set of node features ${\mathscr{X}}_g$ through a sequence of *L* hidden ANN layers, where at each layer $l \in \{1, \ldots , L\}$, the feature vector $x_v$ of each node $v \in {\mathscr{V}}_g$ is updated as:

15 $$\begin{aligned} x_v^l = \mu _l \langle x_v^{l-1}, \{x_u^{l-1}, e_{u, v}\} \rangle \end{aligned}$$

where $\mu _l \langle \cdot \rangle$ is a parametric combination function (operator) with learnable parameters that are updated by the objective (loss) function’s gradients through the ANNs’ backpropagation. The variable $u \in {\mathscr{V}}_g, u \ne v$ is a neighboring node of *v* within *g*, such that *u* and *v* are connected by edge $(\overrightarrow{u, v})$, and $e_{u, v} \in {\mathscr{A}}_g$ is the attribute of edge $(\overrightarrow{u, v})$. When $l=1$, $x_v^{l-1} = x_v^0$, which denotes the original node features ${\mathscr{X}}_g$. After all *L* layers, the resulting output feature $x_v^L$ is the node embedding of the original graph *g*. This work utilizes the local extremum operator (LEConv) proposed in^30^ for $\mu _l$, which finds the importance of nodes with respect to their neighbors using the difference operator, and thus benefits from the distinct edge weight of directed edges. The combining function in Eq. (15) for LEConv is expressed as:

16 $$\begin{aligned} x_v^l = \psi _l \langle \Theta _1^{l-1} x_v^{l-1} + \sum _{(\overrightarrow{u, v}) \in {\mathscr{E}}_g} e_{u,v} (\Theta _2^{l-1}x_v^{l-1} - \Theta _3^{l-1}x_u^{l-1}) \rangle \end{aligned}$$

where $\psi _l \langle \cdot \rangle$ represent the activation function of layer *l*, which is a configurable hyperparameter providing nonlinearity, while $\Theta _1^{l-1}, \Theta _2^{l-1}$, and $\Theta _3^{l-1}$ denote different learnable parameters.

The main objective of cell-switching is to find the optimal strategy $\Gamma _t$ at every time slot to determine the best set of SCs to switch on or off to increase energy efficiency. Therefore, the node features after node embedding will be passed through a final output layer with another parametric function that maps $x_v^L$ to binary values $\gamma _v \in \{0, 1\}$, while this function needs to be continuous to calculate gradients for GNN’s backpropagation. The solution is to first have a function $\Psi \langle \cdot \rangle$ that maps $x_v^{L}$ to the continuous values ranging between [0, 1] to provide the final output of the GNN, followed by another function mapping such continuous GNN output values to binary ones. In practice, $\Psi \langle \cdot \rangle$ can be implemented using another NN layer whose activation function has an output range of [0, 1], and hence is another configurable hyper-parameter of the computation model for the GBCSS. The value discretization can be achieved by the indicator function $I_{[0.5, 1]} \langle \cdot \rangle$ that near-evenly maps the continuous values from [0, 1] to binary values $\{0, 1\}$.

### Complexity

As the problem given in (11) is an NP-hard problem, it does not have a deterministic polynomial-time solution. However, since it is a combinatorial optimization, its optimal solution can be found with an exhaustive search algorithm which iterates through every possible option in the search space. Therefore, it is highly computationally demanding, and since in the cell switching problem has every SC has two possible states (ON and OFF), the total number of state combinations is $2^{N_{SC}}$ which is the steps required for the exhaustive search to find the optimal ON/OFF switching combination.

In contrast, the presented graph representation modeling and GNN computation model aims to reduce the overall computational complexity. With both graph representation and GNN computation model introduced in previous sections, the forward inference of GBCSS procedure is summarized in Algorithm 1, which is a high-level abstraction of the actual implementation using previously introduced notations, to mainly help analyze the algorithm’s complexity.

**Algorithm 1**: Feed-forward inference for the proposed GBCSS at time slot *t*
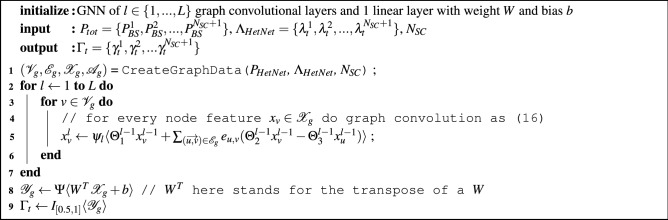


Step 1 of the algorithm denotes the graph data creation according to the graph representation design, which can be treated as a linear function that maps the input $P_{HetNet}$ and $\Lambda _{HetNet}$ to graph-structured data, hence its overall operation steps grows linearly to $N_{SC}$. Step 2 to 8 mimic the computational flow of the GNN computation model and step 9 represents the value discretization introduced in the above section. Step 2 to 7 represents the graph embedding using the LEConv convolution operator and has in total $L \times (N_{SC} + 1)$ operations. Step 8 indicate the linear output layer which essentially performs a linear transformation before passing to the activation function $\Psi \langle \cdot \rangle$, while step 9 simply pass the output of step 8 to the indicator function $I_{[0.5, 1]} \langle \cdot \rangle$ to produce binary output $\Gamma _t$. Both activation functions apply to the input element-wise so their total number operations grows linearly to $N_{SC}$.

Define $\textbf{N} = N_{SC} + 1$ being the total number of BSs in a HetNet unit and thus the number of nodes in the graph representation. The complexity of exhaustive search is then $O(2^{\textbf{N} -1})$ according to the above discussion. In comparison, most operations occur during step 2 to 7 for the GBCSS which is $L \times n$, with other operations being linear. Moreover, the number of neurons for all NN layers will also impact on the overall number of operations. However, *L* will be a constant for a defined GNN model, and each NN layer’s number of neurons will also be a constant upon definition. Therefore, GBCSS’ complexity is bound to $O(\textbf{N})$, which is linear to the total number of BSs in a HetNet unit as introduced in the graph representation. Therefore, this complexity will not lead to a large computational burden compared to the Exhaustive Search algorithm with $O(2^{\textbf{N}})$ that grows exponentially with respect to the total BS number.

### Training and loss function design

The parameters of the GNN computation model can be trained in either supervised or unsupervised learning manner^22^. For supervised learning approach, it is essential to obtain high-quality labelled samples indicating the optimal cell-switching decisions for each input graph *g*. However, exhaustive search that always generates the optimal solutions has the complexity of $O(2^{\textbf{N}})$, and hence it is impractical to generate a dataset with sufficient optimal cell-switching samples as the node size increases (e.g. above 20 nodes). In contrast, other methods cannot always guarantee to produce optimal cell-switching decisions for labelled samples, which may hinder the overall learning performances.

Therefore, this research proposed an unsupervised learning approach to train the proposed GBCSS. Assuming a batch of *B* unlabelled samples of a HetNet unit’s graph representation *g*. The designed loss function ${\mathscr{L}}$ is given by

17 $$\begin{aligned} {\mathscr{L}} = -\frac{1}{B}\sum _{i=1}^{B} \zeta _{j, g} \end{aligned}$$

where $\zeta _{j,g}$ is the objective function for the *j*th sample of graph *g* in the data batch. ${\mathscr{L}}$ aims to directly tune the GNN model to optimize the objective functions in Eqs. (11) and (12). Derived from the calculation of $\frac{P_{tot}(\Gamma )}{\Lambda (\Gamma )}$, $\zeta _{j,g}$ indicates the system-wise power consumption per unit traffic load for the graph representation after cell-switching, following the cell-switching decisions from the GNN outputs. The calculation of $\zeta _{i, g}$ is given by:

18 $$\begin{aligned} \zeta _{i,g}(\Psi \langle x_v^L \rangle ) = \sum _{v \in {\mathscr{V}}_g} \frac{\hat{x}_v}{\hat{a}_{v, 1}} \end{aligned}$$

where $\zeta _{j,g}(\Psi \langle x_v^L \rangle )$ denotes the loss $\zeta _{j,g}$ following the cell switching decision represented by the GNN output $\Psi \langle x_v^L \rangle$; $\hat{x}_v$ and $\hat{a}_{v, 1}$ are the node and edge features after cell-switching, following the calculation of $P_{tot}$, $\hat{\lambda }$ and $\Lambda$, as described in the problem formation and graph representation. Note that $\hat{a}_{v, 1}$ is used instead of a general notation $\hat{a}_{v, u}$ since all edges are defined to connect an SC node to the MC node at index 1, according to the proposed graph representation. The system requirement that the MC should always be switched on is also learned by the GNN, as the magnitude of ${\mathscr{L}}$ will become very large when the output label of the MC node is OFF, due to a substantial decrease of $\Lambda$.

## Evaluation configurations

This section covers the experimental setups and related configurations of the performance evaluation for the proposed GBCSS. The experiments use the EARTH power consumption model^25^ and compares the performance of GBCSS with other cell-switching benchmarks under various metrics. The power consumption characteristics for each types of BSs are summerized in Table 1. For a real-world CDSA HetNet cell-switching scenario, it is natural to consider a set of BSs at fixed geographic locations that experience traffic variances at different time slots of a day and across different days, which is an essential assumption for the experimental configurations in this paper.

For the deployment of GBCSS, it is assumed that the algorithm is implemented at the local controller located at the MC for every HetNet unit in a locally centralized manner, along with all other benchmarking algorithms. At each time slot *t*, all SCs in operation send their factorized traffic load and power consumption measurement to the MC via the backhaul for cell switching measurement, while that of the MC will be directly available at the controller due to where it is deployed. For sleeping SCs, the traffic load will naturally be 0, and the power consumption will be the sleeping power for their corresponding BS types, which is known at the MC upon initial deployment. The “measurement” from sleeping SCs can be filled by the MC after receiving all operating SCs’ measurement reports. As this paper focus on algorithmic design and evaluation, more detailed real-world deployment setup is beyond the scope of this work. Also note that an ultra-dense HetHet may comprise many HetNet units, each consisting of one MC and various numbers of SCs, therefore the obtained results may also be utilized to infer other HetNet units’ performances pattern in the network.

The experiments have been implemented via Python 3.9 using scientific and data analysis libraries Numpy^31^, Scipy^32^, and Pandas^33^, with related result visualizations generated via Matplotlib^34^.

**Table 1:** Power profiles for each type of BSs^25^.

BS type	Power consumption (W)			$\Delta _p$
	Operational $P_o$	Transmit (max) $P_{max}$	Sleep $P_s$	
Macro	130	20	75	4.7
RRH	84	20	56	2.8
Micro	56	6.3	39	2.6
Pico	6.8	0.13	4.3	4.0
Femto	4.8	0.05	2.9	8.0

### Dataset and experimental setups

#### The original dataset

When calculating power consumptions using Eq. (2), it is important to obtain the traffic load $\lambda$ for every BS, and it is also important to evaluate ML-based algorithms using standardized datasets and/or simulation environments^10^. Both aspects considered, an established multi-source dataset^23^ is chosen for the performance evaluation, in which the city of Milan is divided into 10,000 square-shaped grids of 235 m $\times$ 235 m. The grid indices are calculated as $ID_{gird} = (x + 1) + 100 \cdot y$, where $x, y \in [0, 99]$ are for indexing purpose only. More detailed grid information can be found in Fig. 2 of the original paper^23^ and using the grid dataset^35^. In particular, the telecommunication dataset of the Milan city based on real-world call detail records (CDR) data provided by Telecom Italia is used for the evaluation experiments^36^. The dataset contains phone call, text message, and Internet activities between a user and a BS, which are spactially aggregated into each grid according to the spatial intersections among the grid and nearby BSs’ coverage. Additionally, the CDR data was recorded in 10-minute resolution for a two-month period from November 1st, 2013 to January 1st, 2014. Therefore, the original dataset contains 7 types of features: the grid ID, timestamp (representing date and time), in/out Short Message Service (SMS) activities, in/out call activities, and Internet activities. In total, the dataset contains 62 days’ data with 144 time slots per day for 10,000 grids, resulting in 8928 entries of {*in-SMS, out-SMS, in-call, out-call, Internet*} per grid. Although the dataset consists of unitless values (due to commercial confidentiality) for each type of activities, while no information is provided to reverse the spatial aggregation, these activity levels represent the volume of user-network interaction at each time slot and can hence be utilized to calculate and compare traffic loads between grids.

#### Dataset pre-processing and scenario setups

The experiments consider a scenario of a HetNet unit located at the city center area, with different numbers of SCs $N_{sc} \in \{4, 8, 12, \ldots, 32\}$ with BS types assigned uniformly, to evaluate the scalability of GBCSS. In the data pre-processing phase of the evaluation process, CDR values of all activity types are first combined into sum CDR activity data for each grid in the Milan dataset as the cell switching problem considers BSs’ overall traffic loads. This operation fuse the original feature set {*in-SMS, out-SMS, in-call, out-call, Internet*} to a new feature type *sum-load* for each time slot per grid. After this step comes a grid selection and sum CDR value normalization phase to produce factorized values that represent $\lambda$ of BSs. The CDR normalization scale is determined by $\phi$ for each type of BSs, after which the *sum-load* feature of a grid becomes $\lambda _t^n$, $1 \le n \le N_{Sc}+1$ while preserving the traffic variation pattern.

The 10,000 grids in the Milan dataset cover both urban and sub-urban areas containing markedly different behavioural signatures regarding traffic variation trends, as shown in Fig. 7 in the original paper^23^ of the Milan dataset. Therefore, choosing grids within the full grid list may violate the above scenario assumptions. Therefore, this paper focuses on grids around the city center of Milan (Grid 5060 with $x = 59$ and $y = 50$, representing the area around the *Duomo di Milano* cathedral) as defined in the original article^23^. Grids of $x \pm 4, \ y \pm 4$ from Grid 5060 have been chosen, forming an area of 2115 m $\times$ 2115 m that contains 81 grids within the same geographic regions of the Milan city center. This also suggests that SC offloading is feasible in this area within the coverage of the MC, which is a corner stone for cell-switching operations.

By non-repetitive random selections, 12 dates were chosen from the business days within the two-month period in the Milan dataset for all $N_{sc}$ cases, data for 8 of these days were used to form a training dataset^37^, while the other 4 days’ data were utilized to form a validation dataset^37^. Additionally, two dates have been initially preserved before the above random selections, forming a test dataset^37^ that is not used in the training process. Consisting of data from one workday (Nov. 15th, 2013) and one holiday (Jan. 1th, 2014), the test dataset is used for one-day performance evaluation emulating online execution after the algorithm’s deployment, to test the proposed GBCSS’ generalibility. As a result, for the processed dataset after grid assignment, the feature set at time slot *t* is $\{\lambda _t^1, \lambda _t^2, \ldots, \lambda _t^{N_{SC} + 1}\}$ of size $N_{SC} + 1$. The training set contains 1152 entries while the validation and test sets consist of 576 and 288 entries, respectively.

As for the grid assignment, the MC was always assigned with Grid 5060’s normalized activities in each $N_{sc}$ case, while one grid for each SC was then selected non-repetitively within the defined region. Only one round of grid selections was carried out for every $N_{sc}$ case (i.e. every data sample in the generated datasets were from the same set of grids for each $N_{sc} \in \{4, 8, 12, \ldots 32\}$ case). A fixed random seed is used for all $N_{sc}$ cases to provide consistent and reproducible results. After grid assignment, a BS will experience the traffic variation following that of the assigned grid when no cell switching is applied. For readers interested in this temporal aspect of traffic variations, Fig. 5 in the original paper^23^ has provided such information over a week time.

With all experiment setups introduced, the corresponding experimental parameters are summerized in Table 2. All BSs are assumed to have the same maximum capacity in terms of radio resources (bandwidth and resource blocks) to simplify the traffic load normalization during the data pre-processing, and the calculations in Eqs. (12) and (21). The reason is that we are only interested in whether the original traffic load is preserved for each cell-switching scheme according to the introduced performance metrics, following the optimization constraint defined by Eq. (12). Moreover, setting different capacities for each BS type only influences $\phi$ and thus some numerical results for $\Lambda$ and $P_{tot}$ after offloading, while such differences do not influence how a cell switching strategy is formulated.

**Table 2:** Experimental configurations.

Parameters		Values
Number of time slots per day		144
Number of grids considered for each BS		1
Number of days	Training set	8
	Validation set	4
	Test set	2
		(1 workday & 1 holiday)
Bandwidth; number of resource blocks for BSs		20 MHz, 100

### GNN setups

For the experiments, the dataset goes through the graph representation process and the normalized load factors become the node features $\mathcal (X_g)$ and the calculated power consumption for all nodes become the edge features ${\mathscr{A}}_g$.

Some configurable hyper-parameters are mentioned. For the evaluation, $L = 3$ hidden layers for node embedding in the GNN computation model are configured, with the neuron size or 128, 128 and 64. The activation function $\mu \langle \cdot \rangle$ is set to the Rectified Linear Units (ReLU)^38^ for all 3 hidden layers. For the output layer setup and binary value translation, $\Psi \langle \cdot \rangle$ is configured as:

19 $$\begin{aligned} \Psi \langle x_v^L \rangle = \sigma \langle W^T x_v^L + b \rangle \end{aligned}$$

where *W* and *b* represent learnable parameters (weights and biases) of a linear transformation, *T* denotes the matrix transpose, and $\sigma \langle \cdot \rangle$ is the sigmoid activation function^39^. This makes the GNN output continuous values between [0, 1], which can then be used to provide binary via $\gamma _v = I_{[0.5, 1]} \langle \Psi \langle W^T x_v^L + b \rangle \rangle$ as previously discussed.

For other GNN configurations, the batch size is set to 64, and each GNN model (one for each $N_{sc}$ instance) is trained for a maximum epoch of 200 in the experiments. The learning rate (LR) is initially set to $10^{-3}$, with a dynamic LR scheduler^40^ configured which reduces the LR by a factor of 2 if no improvement has been made to the loss defined in Eq. (18) for the past 10 epochs. The optimization algorithm is set to the “Adam with decoupled weight decay” (AdamW) optimizer^41^. The above GNN configurations are summerized in Table 3. The GNN model and other deep learning related implementations are fulfilled by Pytorch^42^ and Pytorch Geometric^43^.

**Table 3:** GNN configurations.

Hyperparameters	Values
Number of hidden layers; Neuron size	3; 128 $\times$ 128 $\times$ 64
Hidden layer activation function	ReLU^38^
Output layer activation function	Sigmoid^39^
Optimizer	AdamW^41^
Learning rate (LR)	$10^{-3}$
LR scheduler	Reduce LR on Plateau^40^
Batch size	64
Maximum number of epochs	200

### Benchmarks

Benchmarks are necessary to compare the performance of the proposed GBCSS, and the comparison basis was selected following this rationale: 1. The optimal solution (where applicable) maximizing energy saving while preserving all original traffic, which stands as the performance upper bound. 2. The bottomline without any cell switching strategy, such that all BSs’ traffic and thus user QoS are preserved while sacrificing the energy efficiency optimization. 3. Another sub-optimal cell switching solution whose performance can be directly compared with GBCSS in terms of the performance metrics defined in the next section. As a result, three different methods are used for benchmarking, introduced as follows:

- **Exhaustive search (ES)**: This method iterates through all possible combinations of binary switching options consisting of the on/off states for all SCs. It also considers the available radio resources at the MC for offloading such that the maximum traffic demand that the network can serve is not exceeded during power consumption optimization. Therefore, this method checks all possible SC combinations to switch off, and guarantees to produce the optimal cell-switching policy that minimizes the total power consumption of the network while preserving the user QoS in the network.
- **Linear function approximation-based SARSA (FA)**: This is a state-of-the-art RL-based cell-switching scheme proposed by Ozturk et al.^12^. FA defines every time slot *t* as an episode, and uses a feature vector $\{P_{tot}, \lambda _t^1\, \lambda _t^2, \ldots, \lambda _t^{N_{SC}+1}\}$ containing all BSs’ load factors and system-wise power consumption to train a parameter set $\theta$ that represents the optimal cell switching policy via linear function approximation. For interested readers, more detailed design of the FA algorithm can be found in the original paper.
- **All-on**: As its name indicates, this approach implements a scheme with no offloading and cell-switching, and hence all BSs are always left ON. This method ensures the user QoS within a HetNet unit, but no energy saving can be achieved since no SCs will be switched off. It is used as the baseline of optimal throughput with respect to the power consumption bottomline.

### Performance metrics

This subsection introduces the metrics used to evaluate the performance of GBCSS compared with the selected benchmarks. As all metrics are based on the dataset, they are chosen as:

- **Power consumption** $P_{tot}$: This is the HetNet unit’s instantaneous power consumption during a day defined in Eq. (1) for each method calculated based on Eq. (2). Measured in Watts (W), this metric evaluates the performance of each solution as it reflects the variations in network power consumption in different time slots of the day.
- **Total energy saved** $E_{saving}$: The total energy saved is another straightforward yet essential metric to assess the performance of GBCSS. Compared to the All-on method that does not consider energy-saving, it is calculated as $E_{saving} = E_M - E_{ON}$, where $E_{ON}$ and $E_M$ are the total energy consumption with All-on method and with one of the cell-switching solutions: exhaustive search, the FA-based solution and GBCSS, such that $E_M \in \{E_{GNN}, E_{ES}, E_{FA}\}$. The calculation of daily total energy consumption *E* for each method following the dataset time slots as follows:
  20 $$\begin{aligned} E = \sum _{t=1}^{N_{slots}} P_{tot}^t \times 60 \times 10 \end{aligned}$$
  where $P_{tot}^t$ is the power consumption (W) of the HetNet unit at time slot *t*. As *t* is presented in 10-minute resolution in the Milan dataset, one day (24 hours) leads to $N_{slots} = 144$. Additionally, since the evaluation process may include multiple day samples, the average values among different day samples are further calculated to represent *E* in such cases.
- **Normalized network traffic load** $\Lambda _{\%}$: This metric is the HetNet unit’s sum traffic load after offloading normalized by that before offloading. As the All-on method does not implement any offloading and cell-switching schemes, thus can always preserve the original traffic loads. This metric can hence be calculated as $\Lambda _{\%} = \frac{\Lambda _{M}}{\Lambda _{ON}}$, where $\Lambda _M \in \{\Lambda _{GNN}, \Lambda _{ES}, \Lambda _{FA} \}$ is the sum traffic load after offloading using the covered solutions and $\Lambda _{ON}$ is the sum traffic load using the All-on method. Following Eqs. (4)–(5) and (12), the sum traffic load of one day (24 hours) using any of the covered solutions is calculated as:
  21 $$\begin{aligned} \Lambda = \sum _{t=1}^{N_{slots}} \left( \hat{\lambda }_t^1 + \sum _{i=2}^{N_{SC}+1} \phi _i\hat{\lambda }_t^i\right) \end{aligned}$$
  where $N_{slots} = 144$ in the Milan dataset for 10-minute time slots.
- **Normalized energy efficiency** $\eta _{\%}$: This is the daily energy efficiency of the HetNet unit implemented cell-switching solutions, normalized by that without cell-switching (i.e. All-on). Similar to that of $\Lambda _{\%}$, this relative energy efficiency is calculated as $\eta _{\%} = \frac{\eta _{M}}{\eta _{ON}}$, where $\eta _M \in \{ \eta _{GNN}, \eta _{ES}, \eta _{FA} \}$ is the energy efficiency of the HetNet unit using the corresponding cell switching solution while $\eta _{ON}$ is that without cell switching. The energy efficiency $\eta$ using any of the covered solution is calculated as:
  22 $$\begin{aligned} \eta = \frac{\Lambda }{E} \end{aligned}$$

## Results and discussions

Following the evaluation setups, this section covers the experimental result analysis for the proposed GBCSS, compared with other benchmarks. Qualitative discussions regarding GBCSS with some state-of-the-art solutions are also included in this section.

For learning-based solutions (GNN and FA), an offline training stage was first carried out. The trained GNN and FA’s policy were then exported to produce statistical results (i.e. metrics $E_{saving}$ and $\Lambda _{\%}$ with respect to $N_{sc}$) using the validation dataset. Finally, the two day samples in the test dataset is used to emulate the online deployment for cell-switching execution that provides results for $P_{tot}$ throughout the day (24 h). Unless otherwise stated, the results for each $N_{sc}$ case are generated using the GNN trained with the dataset generated for that case. Note that during the online execution phase, it is possible to update the learning models using the latest collected data to further improve the models’ performances. However, such online model updating is beyond the scope of this paper.

Before presenting the results regarding each metric, it is also important to analyze the convergence behaviors of the GNN training. Using the configured GNN setups, the loss function value defined in Eq. (18) was collected during the training stage. For all considered $N_{sc}$, the GNN model managed to converge within the first 20 epochs for 7 out of 8 $N_{sc}$ cases, with the minimum epochs for convergence being 5, and the maximum epochs around 55. As the loss records for all 8 $N_{sc}$ cases cannot be summarized clearly in a graphical manner, the essential information has been presented above.

### Statistical results from validation set

**Figure 3:**
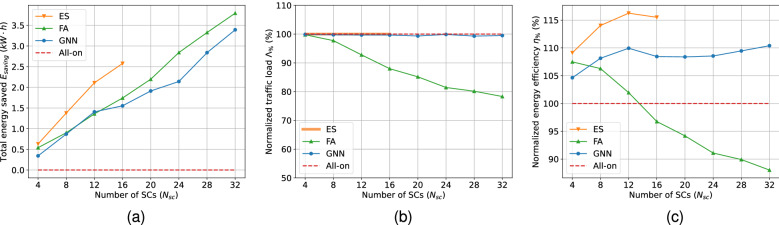
Statistical results from the validation set for different $N_{sc}$ (**a**) Total energy saved $E_{saving}$. (**b**) Relative traffic load $\Lambda _{\%}$. (**c**) Relative energy efficiency $\eta _{\%}$. ES is not executed for $N_{sc} > 16$ due to huge time consumption.

Figure 3 shows the results of metrics $E_{saving}$, $\Lambda _{\%}$, and $\eta _{\%}$ with respect to $N_{sc}$. The average values using the 4 day samples in the validation dataset are calculated for the metrics. It is noteworthy that the ES algorithm has only been executed for $N_{sc} \in \{4, 8, 12, 16 \}$ due to time consumption burden as the algorithm is highly computationally demanding with a complexity of $O(2^{\textbf{N}})$. This means that the processing time for the ES algorithm doubles for every unit $N_{sc}$ increment. In contrast, GBCSS learns to find a sub-optimal solution that approximate to the optimality as much as possible while maintaining a much lower computational complexity of $O(\textbf{N})$.

The metric $E_{saving}$ is the optimization objective for cell switching solutions according to the problem definition in Eq. (11), and is an essential metric to consider. It can be seen in Fig. 3a that the daily total energy saved increases when $N_{sc}$ is raised for all cell-switching methods, based on the fact that deploying more SCs leads to increased power consumption, while creating more possibilities for offloading and cell switching when the MC has sufficient resource to take over, and hence larger energy saving.

For $N_{sc} \in \{4, 8, 12, 16\}$, the saved energy using the ES algorithm is the highest among the considered solutions, and can be expected to remain so for larger $N_{sc}$ values if ES was to be executed. For GBCSS, the energy saved is lower than that of ES. For $N_{sc} \in \{4, 8, 12, 16\}$, the GBCSS achieves 53.97%, 63.04%, 66.82%, and 60.08% of ES’ $E_{saving}$ performance, resulting in a 62% $E_{saving}$ performance for the 4 $N_{sc}$ cases. Moreover, the GNN is able to further increase the $E_{saving}$ for a large number of deployed SCs as the slope of the $E_{saving}$ curve has clearly increased for $N_{sc} \in \{24, 28, 32\}$. The detailed discussion regarding this aspect is covered in the one-day performance analysis with more supporting results.

Interestingly, the $E_{saving}$ using the FA benchmark is clearly larger than that of GBCSS for most considered $N_{sc}$ cases except for $N_{sc} = 8$ and 12, in which both solutions result in similar $E_{saving}$. GBCSS can achieve a maximum 103.61% and a minimum of 62.28% $E_{saving}$ performances compared with using the FA, with an average of 86.60% $E_{saving}$ performance compared with using the FA for all $N_{sc}$ cases. This suggests that the FA benchmark outperforms GBCSS in raw energy saving.

However, it is equally important to also consider the metric $\Lambda _{\%}$, which indicates how much of the original traffic load without cell switching (i.e. All-on) can be preserved using different cell-switching solution and represents the optimization constraint defined in Eq. (12). According to its definition, the maximum value for $\Lambda _{\%}$ is 100%, which means that all original traffic load is preserved after cell switching execution.

Figure 3b shows this metric with a reference red dashed line of the All-on method stands for the upper bound. It can be seen in the figure that ES has $\Lambda _{\%} =100\%$ for $N_{sc} \in \{4, 8, 12, 16\}$, and is reasonable to assume this trend will be consistent for other $N_{sc}$ cases. In comparison, using the proposed GBCSS results in an average $\Lambda _{\%}$ of 99.63% for all 8 $N_{sc}$ cases, with a maximum of 99.88% and minimum of 99.31%. This suggests that the GNN learns to preserve the user QoS as much as possible when reducing the HetNet unit’s energy consumption.

In contrast, it can be seen that the $\Lambda _{\%}$ using FA decreases from 99.77% for $N_{sc} = 4$ to 78.30% for $N_{sc} = 32$. This means that compared to GBCSS, the extra energy saved when using the FA benchmark as shown in Fig. 3a will cost 21% of the original traffic load and hence the user QoS in the worst case. The reason is that using the offline trained FA algorithm for online decision making leads to much more frequent decision making that causes the MC to overload and thus user QoS downgrade, as only the MC can take over the traffic load of a SC according to the problem formation.

Considering both energy consumption and traffic loads, Fig. 3c shows the normalized daily energy efficiency $\eta _{\%}$ for the considered cell switching solutions with respect to All-on. It is clear that $\eta _{\%}$ of using the ES algorithm is the highest and achieves an average $\eta _{\%}$ of 13.74% among the $N_{sc}$ cases, with a maximum energy efficiency gain of 16.25% compared to that of All-on, while $\eta _{\%}$ using the FA solution drops continuously and becomes even lower than that of All-on due to a large proportion of original traffic load being sacrificed to achieve higher power saving. In comparison, GBCSS achieves an average and maximum $\eta _{\%}$ of 8.50% and 10.41% respectively compared to All-on. The trend of $\eta _{\%}$ using GBCSS is similar to that of ES based on the results for $N_{sc} \in \{ 4, 8, 12, 16\}$ according to Fig. 3c, while overall the energy efficiency gain using the GNN is about 62% for these $N_{sc}$ cases. Moreover, assuming the average $\eta _{\%}$ (13.74%) using the ES is preserved for $N_{sc} in \{20, 24, 28, 36\}$, the GNN can achieve a maximum 75.76% of ES’ performance regarding energy efficiency gain.

Nevertheless, the FA benchmark still outperforms the proposed GBCSS when $N_{sc} = 4$ with FA’s $\eta _{\%}$ being around 2.5% larger as in Fig. 3c. A potential reason is that the GNN is not able to further approximate to the optimal solution when the gradient calculated via the loss function Eq. (18) becomes too small, as learning to always switch on the MC leads to a large ${\mathscr{L}}$ improvement when training the GBCSS. In comparison, the FA benchmark avoids such situation as the action for the MC has predefined to be always ON. However, the relative underperformance of GNN in this case can be regarded as insignificant as the overall energy saved in this case is low due to only 4 SCs were deployed.

### Test set performance results

The results generated with the test dataset for one-day power consumption using each solution are presented for 3 $N_{sc}$ cases (i.e. $N_{sc} \in \{ 4, 12, 32\}$) that represents scenarios of a small, medium and large number of deployed SCs within the considered $N_{sc}$ cases. The results of node size generalization test for the GNN is also covered in this section.

**Figure 4:**
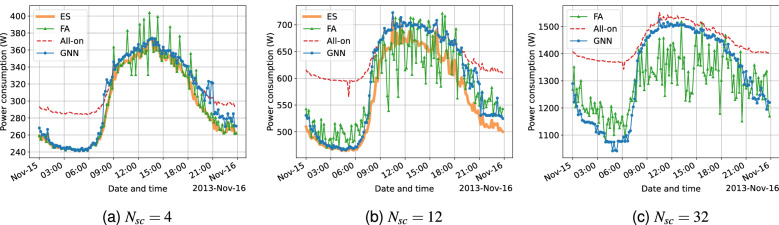
One-day performance results for the workday sample (Nov. 15th, 2013) in the test set with respect to power consumption for different $N_{sc}$.

#### Performance comparison on workday samples

Figure 4 shows the power consumption per time slot using GBCSS and other benchmarks throughout a workday (from 00:00 a.m. to 11:59 p.m.) for the three $N_{sc}$ cases. Due to the same computational complexity reason as for statistical results analysis, the ES algorithm is not executed to generate results for $N_{sc} = 32$.

According to Eqs. (2) and (3), the power consumption calculation is a linear transformation of $\lambda$ when no BS is put into sleep. Therefore, a HetNet unit’s traffic load trend throughout a day can be inferred by the power consumption trend of the All-on method. It can be seen in Fig. 4 that the HetNet unit’s power consumption is relatively low before dawn with only a small number of active users, while the traffic load starts to rise around 8 a.m. and peaks before midday, leading to an increased power consumption period with less potential for power saving. Later, the traffic load start to decline more significantly in the late afternoon (4 p.m.), leading to another period for energy efficiency optimization using cell switching.

As shown in Fig. 4a, all 3 cell-switching solutions are able to significantly reduce the power consumption from 0 a.m. to 8 a.m.. During this period, the power consumption using GBCSS highly mirrors the behavior of the ES algorithm. During the high-traffic hours, GBCSS turns to follow the strategy of All-on, which is a suboptimal strategy for this time period. From 4 p.m. until midnight, the GNN also learns to reduce the HetNet unit’s power consumption, but the performance is not as significant as it does in the time period before dawn compared to the optimal results computed via ES. In contrast, the FA benchmark also mirrors the behavior of ES over the day, and overall outperforms GBCSS especially after 4 p.m.. Moreover, during the busy hours between 9 a.m. and 4 p.m., it can be seen that for some time slots, the power consumption of using the FA benchmark becomes less than that using ES. Because ES produces the optimal cell switching decisions for power saving while maintaining the original traffic loads in the HetNet unit, it can be inferred that FA’s further power-saving comes from sacrificing the user QoS.

For the $N_{sc} = 12$ case in Fig. 4b, the behavior of the ES algorithm remains the same as in the $N_{sc} = 4$ case, while a larger gap can be found compared with the power consumption of All-on, suggesting a larger potential for energy efficiency optimization. Similarly, GBCSS also demonstrates comparable results consistent to those in Fig. 4a, with the performance after 4 p.m. also improved compared to that in the $N_{sc} = 4$ case. However, the results of the FA benchmark start to have more significant fluctuations in Fig. 4b, with obviously lower power consumption compared with using the ES during the busy hours. Combining with the results in Fig. 3b, this means that the FA benchmark starts to output more decisions that causes user QoS sacrifices.

**Figure 5:**
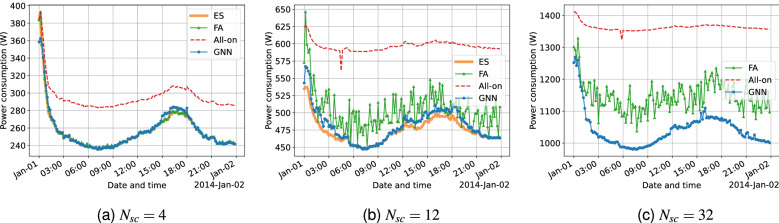
One-day performance results for the holiday sample (Jan. 1st, 2014) in the test set with respect to power consumption for different $N_{sc}$.

As for the $N_{sc} = 32$ case in Fig. 5c, the fluctuation in the results of the FA benchmark has even worsen with the number of decisions sacrificing the user QoS further rises. An obvious explanation to this trend is that the FA benchmark utilizes the linear function approximation technique to represent the value function, which may not have enough expressiveness for scenarios with higher complexity. In contrast, GBCSS shows much more stable results that is consistent to those for $N_{sc} = 4 \,{\text {and}}\, 12$. Moreover, GBCSS also starts to switch off SCs during the busy hours, and the power consumption during this period becomes smaller than that of All-on for $N_{sc} = 32$ according to Fig. 4c. This is much more similar to the strategy that ES produces based on results in Fig. 4a,b. As discussed in the above section, the main reason to it can be that the loss function cannot be significantly optimized when $N_{sc}$ is small, following the calculation in Eq. (18). Moreover, cell switching during a time period with intensive traffic mainly results in marginal power consumption improvement for small $N_{sc}$, as shown by the results using the ES algorithm. In contrast, a larger $N_{sc}$ leads to more potential for a significant loss reduction during the busy hours. This can be regarded as an advantage to exploit, because the envisioned ultra-dense HetNet development for beyond 5G will result in significantly large numbers of SCs to be deployed, where the GNN may find great potential in approximating to the optimal cell switching decision. All the results presented in this section so far correspond to the discoveries in Fig. 3.

Additionally, it can be seen in Fig. 4 that sometimes using GBCSS and the FA benchmark results in more power consumption than using the All-on method during the busy hours for $N_{sc} = 4$ and 8. This raises another question as it is counter-intuitive to have such observations that switching off some BSs causes more power consumption than always keeping all the SCs on. However, considering Eq. (2) together with the parameters in Table 1, it is possible for certain cell switching decisions to cause an overall larger power consumption by offloading to the MC. For example, switching off a half-loaded femto BS results in a 2.1W power consumption reduction under the experiment configuration, but the MC taking over the offloaded traffic (assuming sufficient resource) will have its power consumption raised by 47W, which leads to a -44.9 W power consumption gain. A formal mathematical proof can be found in^12^ that uses the same power model and BS power profiles.

In summary, the proposed GBCSS is able to closely approximate the optimal cell switching decisions computed by the ES algorithm when the total traffic load on the HetNet unit is low, while tends to generate a suboptimal strategies during the time period with intensive traffic. Nevertheless, such suboptimal strategy during the busy hours can be improved when $N_{sc}$ becomes larger (Fig. 4c), when the GNN starts to mirror the behaviors of ES as in Fig. 4a,b. The one-day performance evaluation on a workday produces results that closely correspond to the statistical results generated from the validation dataset.

#### Performance comparison on holiday samples

Under the same setup, Fig. 5 shows the power consumption using different cell switching solutions on the New year’s day holiday (2014/01/01). The trending in the figures corresponds with the event of people celebrating the new year’s eve, leading to a large number of active users and hence high power consumption throughout the early hours after midnight. In comparison, the overall power consumption during daytime is more stable compared with that during the workday sample in Fig. 4.

Furthermore, it is clear that using cell switching solutions results in significant power savings during the daytime. This is similar to the two power-saving time periods in Fig. 4, suggesting that during such a holiday, mobile service requests during the normal busy hours are not as intensive compared to that in a workday. Moreover, in Fig. 5a, the power consumption using both GNN and FA is nearly identical to the optimal results using the ES benchmark. In addition, the GNN makes no decisions that cause the power consumption to be higher than All-on and FA also performs significantly better in this regard. The reasoning to this phenomenon is that learning-based solutions learn to capture the power saving potential during low-activity time periods better than during the high-activity periods, combined with the results in Fig. 4.

Other results found in Fig. 5 are highly comparable to the findings in Fig. 4, such as the results using the FA benchmark have fluctuations with the magnitude increases for a larger $N_{sc}$, while the GNN is more stable in this regard. As these aspects are already discussed in the workday case, this section includes no further elaborations.

#### Generalization capability on node size

A remarkable feature of GNN models is their node size invariance, indicating that as long as the data with a similar underlying topology can be expressed using the same graph representation, a GNN model trained using data of node size *i* can be directly use to produce results for node size *j* ($i \ne j$). This feature greatly boosts the generalization capability of GNN models compared with other ML models, leading to a significant cost reduction when deploying GNN models to different scenarios for a defined task.

Therefore, this section presents the node size generalization test to the proposed GBCSS. The workday data samples in the test dataset is used. Two GNN models trained with training data of $N_{sc} = 4$ and 32 are applied in this test, while the node size for the test case is $N_{sc} = 12$ for both models to give a clearer comparison. Because RL-based solutions need to confirm the feature space and/or action space that cannot be naturally extended by the model itself without reapplication, the FA benchmark is hence not applicable in this evaluation.

**Figure 6:**
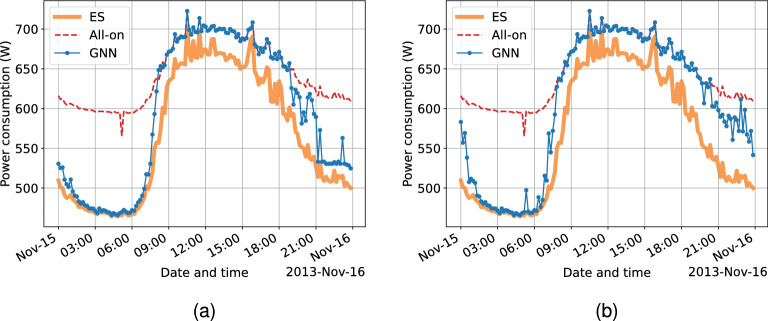
One-day power consumption results for the GNN’s node size generalization test, with models trained using two different node sizes tested with $N_{sc} = 12$. (**a**) $N_{sc} = 4$ for training. (**b**) $N_{sc} = 32$ for training.

The one-day power consumption results of this test is shown in Fig. 6. These results shows that both models trained with different node sizes (both larger and smaller node size during the training stage) can be directly utilized in the $N_{sc} = 12$ scenario. For the two lower-traffic periods, 0 a.m. to 8 a.m. and after 4 p.m., both models generate comparable results to that in the same node size scenarios in Fig. 5b. Furthermore, it can be seen that the models follow some detail from what learned in the original node size scenario. For example, the GNN model trained with $N_{sc} = 4$ produces some sub-optimal decisions that lead to higher power consumption around 9 a.m., similar to that in Fig. 4a, while the GNN model trained with $N_{sc} = 32$ tends to result in large power consumption around 0 a.m., which corresponding to the behavior in Fig. 4c. Unfortunately, the model trained with $N_{sc} = 32$ does not maintain the strategy to switch off some SCs for power saving as in Fig. 4c for $N_{sc} = 12$, while keeps mirroring All-on during the busy hours, similar to that in Fig. 4b. The reason to this may still be the learned loss function characteristics, that a smaller $N_{sc}$ leads to insignificant loss improvement for cell switching during busy hours, as discussed for the workday case.

The node size generalization test results suggest that models trained with one node size can be directly applied to a similar scenario with another node size. Although the performance may not be optimal, this feature can greatly reduce the cost of model transfer, as the whole GNN model can be directly utilized without any preparatory steps. After the transfer, the model can be updated using data collected in the new scenario to learn the underlying patterns to improve performance.

## Conclusion

The development of cellular networks has led to the proliferation of network deployment with BSs being the major energy consumers in cellular networks. This has resulted in calls for greater energy efficiency to meet green and sustainable cellular network demands when applied to real-life network deployments and architectures. As GNN has the significant features of learning graph structured data to improve training robustness, and node size invariance that largely reduce computational cost for redeployment, this paper initially explores a GNN-based cell switching solution (GBCSS) for a CDSA HetNet which can be deployed at each macro BS, and is capable of learning the optimal policy in a dense HetNet environment to save energy and while maintaining the user QoS. The GBCSS approach was then evaluated using the Milan telecommunication dataset based on real-world CDR information and compares it with state-of-the-art benchmarks.

This showed that the GBCSS approach can attain 10.41% energy efficiency gains compared to the baseline power with no cell switching, while maintaining an average of 99.63% of the original traffic loads for differing numbers of BSs, suggesting that virtually no user QoS is sacrificed while reducing energy consumption. This performance is 75.76% of the optimum results computed by the ES algorithm. Additionally, the GNN model trained using data from only workdays generalizes well to both workday and holiday test cases, and is capable of learning the pattern for cell switching during the busy hours in a larger node size (number of SCs deployed) setup for further performance improvement. Node size generalization tests were also performed, with the results supporting the notable feature of GNN’s node size invariance that models trained using data of one node size can be directly utilized in scenarios with different node sizes. Furthermore, GBCSS has a computational complexity of $O(\textbf{N})$ for online execution, and is thus much more scalable compared to the ES and similar algorithms of $O(2^{\textbf{N}})$ as discussed in the complexity analysis.

The proposed GBCSS produces satisfactory energy saving in the network with almost no impact on the user QoS, while showing great potentials for a large number of deployed SCs. Besides, the proposed solution has a very good generalization ability and scalability. All these results make GBCSS a promising candidate for practical cell switching applications to future ultra-dense HetNets. With the development of 6G comes new energy efficiency and network intelligence demand, and the world is also witnessing the rise of energy prices, implementing GBCSS will result in significant energy cost saving, while also relieving the deployment cost of the learning-based algorithm. This will significantly relieve the operational cost for both developed and developing markets.

Future research in this direction includes combing RL algorithms and GNN to further improve the GBCSS’ convergence to the optimality and thus better performance, while exploring heterogeneous graph representation for cell switching, incorporating date and time information in the graph representation to improve robustness for GBCSS also remains of high importance. Moreover, as this work mainly focuses on the algorithmic design, investigating how GBCSS and learning-based algorithms in general may be deployed in a real-world scenario considering detailed protocol stacks is also a valuable direction.

## Data Availability

The original data that support the findings of this study are available in the Harvard Dataverse repository, https://doi.org/10.7910/dvn/EGZHFV. The datasets are from third parties and distributed under the terms of the Creative Commons CC BY license. The original work^23^ has been properly cited in the “Dataset and experimental setups” section of this paper.
